# The impacts of caregiving intensity on informal caregivers in Malaysia: findings from a national survey

**DOI:** 10.1186/s12913-021-06412-5

**Published:** 2021-04-27

**Authors:** Suhana Jawahir, Ee Hong Tan, Yeung R’ong Tan, Sarah Nurain Mohd Noh, Iqbal Ab Rahim

**Affiliations:** Institute for Health Systems Research, National Institutes of Health, Ministry of Health Malaysia, Blok B2, Kompleks NIH, No. 1, Jalan Setia Murni U13/52, Seksyen U13 Setia Alam, 40170 Shah Alam, Selangor Malaysia

**Keywords:** Caregivers, Informal care, Health status, Malaysia, Population health, Health surveys

## Abstract

**Background:**

Provision of informal care may adversely affect health, daily and social activities of the informal caregivers, but few studies have examined these effects in relation to caregiving intensity. This study examined the predictive factors associated with the effects of caregiving roles on health, daily and social activities of informal caregivers, accounting for caregiving intensity.

**Methods:**

Data of adults aged 18 years and over from the National Health and Morbidity Survey 2019 were used. Respondent’s demographic, socioeconomic, health, and caregiving-related characteristics were described using complex samples analysis. Logistic regression analysis was performed to examine the factors affecting health, daily and social activities of caregivers, accounting for caregiving intensity.

**Results:**

Five point one percent of adults in Malaysia provided informal care. High intensity caregivers were more likely to be actively employed and provided longer duration of care compared with low intensity caregivers. For low intensity caregiving, females, those aged 35–59 years, and those with long-term condition were more likely to have negative effects on health. Daily activities of non-Malays were more likely to be affected, while no factor was found significantly associated with effect on social activities. For high intensity caregiving, caregivers aged 60 and over, those received training and those without assistance were more likely to have negative effects on health. Daily activities of those without assistance were more likely to be affected. Social activities of non-Malays, those received training and those providing care for 2 years or more were more likely to be affected.

**Conclusions:**

Our study indicates that both low- and high-intensity caregivers have common features, with the exception of employment status and care duration. Caregiving, regardless of intensity, has a significant impact on caregivers. In order to reduce the negative consequences of caregiving responsibilities, all caregivers need assistance from the community and government, that is customised to their needs. By addressing the factors contributing to the negative effects of caregiving, a continuation of informal caregiving can be sustained through policies supporting the growing demand for informal care necessitated by an ageing population and higher life expectancy in Malaysia.

## Background

Informal care is defined as the provision of unpaid care or assistance to individuals who need help with activities of daily living due to chronic health conditions, disability or old age [1]. Informal care may be provided by family members, relatives, friends or neighbours, but excludes care provided by professionals or through organised volunteer services [2]. Care provided includes: 1) personal care, such as assisting in walking, feeding, dressing, toileting and bathing; 2) healthcare, such as providing transportation to the doctor or health facility and managing medications; and 3) other assistance, such as contributing to financial support, supervision and food preparation [3].

The contribution of informal caregivers in providing care rarely receives recognition and support in Malaysia [4], even though the Malaysian population relies on home-based informal care more than institutionalised care [5]. Family or friends usually provide the first line of support for informal care, as cultural values and norms pertaining to perceived family obligation influence people to care for their next-of-kin [2, 4, 6].

With an ageing population and increased burden of diseases, informal care has become increasingly important in complementing formal professional care to meet future healthcare needs. Globally, governments are increasingly providing support for informal caregiving, which is considered part of the healthcare system [7]. Many developed countries promote the use of informal care to reduce the costs of formal professional care services including healthcare services [2, 7–9]. However, informal caregiving has been shown to contribute to various positive and negative effects [10–13]. The effects of caregiving roles of informal caregivers are widely studied globally [10, 14–17]. Caregivers who spend long hours caring for others are at a higher risk of experiencing negative impacts on their daily life and health [18–20], including forced early retirement [21, 22], reduced working hours [9, 22], limited leisure time [9], increased financial burden [23] and poor health [19, 24, 25].

A previous study conducted in Malaysia revealed that caregiving responsibilities affected the emotional, financial, social and/or physical well-being of informal caregivers [4], though to the best of our knowledge, no published study has used national data to assess the impact of intensity of caregiving in Malaysia. Our study intends to fill the knowledge gap in this area to support planning and policy formulation for providing support to informal caregivers. Policies addressing the needs of informal caregivers are crucial to support them in performing their demanding and challenging roles. Without adequate support, the demanding tasks involved in caregiving may be detrimental to caregivers’ health and well-being.

The objectives of the current study were to: 1) determine the characteristics of caregivers based on care intensity level, 2) examine the predictive factors associated with the effects of caregiving roles on health, daily and social activities of informal caregivers providing a low-intensity level of care, and 3) examine the predictive factors associated with the effects of caregiving roles on health, daily and social activities of informal caregivers providing a high-intensity level of care.

## Methods

### Design and participants

The data for this study were sourced from the National Health and Morbidity Survey (NHMS) that was conducted in 2019 [26]. The NHMS was a cross-sectional population survey with two-stage stratified random sampling among the non-institutionalised population of Malaysia. The stratification was performed by state and urban/rural locality. States and federal territories constituted the primary stratum, and urban and rural areas within the states comprised the secondary stratum. The sampling frame for the NHMS was provided by the Department of Statistics Malaysia (DOSM) using the National Population and Housing Census 2010. Based on the frame, Malaysia was divided into contiguous geographical areas called “enumeration blocks” (EBs). Based on its population size, each EB was defined and categorised into either urban or rural areas by the DOSM. A gazetted area with a combined population of 10,000 or more was categorised as an urban area, whereas a gazetted area with a combined population of less than 10,000 was considered a rural area. All states and federal territories were included in the survey. Within each state, selected number of EBs from urban and rural areas were randomly chosen. Using the probability proportional to size sampling technique, where EBs were the primary sampling unit, a total of 463 EBs, 350 EBs in urban areas and 113 EBs in rural areas, were randomly chosen from the total EBs in Malaysia. Subsequently, second-stage sampling resulted in the selection of 14 living quarters (LQs) in each selected EB. All eligible household members within the selected LQs, equalling a total of 16,688 respondents of all ages who were living in the selected households in the 2 weeks prior to the interviews, were invited to participate in the survey. A detailed methodology and sampling design of the survey is described in the NHMS 2019 technical report [26]. In this study, adults aged 18 years and above with complete key demographic and socioeconomic variables and complete responses to questions related to their health and informal caregiving role were included in the analysis.

### Data collection

Data were collected from July to October 2019 by trained data collectors via face-to-face interviews using a validated questionnaire [27, 28]. The questionnaire was programmed into an application and uploaded onto tablets used as mobile tools to collect data, store and back up data on the SD cards and upload data to the central system. Houses that were vacant or closed during the initial visit were revisited at least three times to maintain the required sample size. Individuals who gave informed consent were invited to join the survey and respond to the questionnaire. The tenets of the Declaration of Helsinki were followed throughout the conduct of the study. Consents from the respondents were obtained before they were interviewed. The survey protocol for NHMS 2019 was approved by Medical Research and Ethical Committee (MREC), Ministry of Health Malaysia [KKM/NIHSEC/P18–2325(11)] and was registered in the National Medical Research Register, Ministry of Health Malaysia (NMRR-18-3085-44,207).

### Study variables

Initially, the respondents were briefed on our definition of informal care – that is, care that was provided for at least 3 months and did not involve wage or salary, community service or volunteer activity. Following this, the respondents were asked to answer “yes” or “no” to the following questions: “In the last 12 months, from ... 2018 till today, did you provide care to your household member with long-term illness, elderly or disabled?” and “In the last 12 months, from ... 2018 till today, did you provide care to other than your household member with long-term illness, elderly or disabled?” For the purpose of analysis, respondents who answered “yes” to either of the questions were categorized as informal caregivers. Level of intensity of care was assessed by the question “In total, how many hours per week did you normally spend providing care to the care recipient?” The number of caregiving hours was then grouped into two categories: 1) 1–19 h, and 2) 20 or more hours.

#### Independent variables

##### Demographic and socioeconomic characteristics

Participant demographic and socioeconomic characteristics included sex, ethnicity, age, education level, marital status, employment status, income and location (urban/rural). Income was calculated based on monthly household income and analysed as quintile before being grouped into three categories: 1) low income (comprised of the first quintile representing the lowest 20% and the second quintile); 2) middle income (comprised of the third and fourth quintiles); and 3) high income (comprised of the fifth quintile representing the highest 20%).

##### Health characteristics

Self-reported health problems, long-term conditions and self-rated health were included as independent variables related to caregivers’ health. For self-reported health problems, the respondents were asked to answer “yes” or “no” to the question “In the last two weeks, did you experience any of the following health problems such as fever, sore throat, difficulty in swallowing, running nose or blocked nose, cough or others?” Long-term conditions were defined as the presence of any non-communicable diseases (diabetes, hypertension or hypercholesterolemia) which were self-reported by the participants as diagnosed by a doctor or healthcare professional. The respondents were asked to answer “yes” or “no” to each of the following questions: “Have you ever been told by a doctor or assistant medical officer that you have diabetes?”; “Have you ever been told by a doctor or assistant medical officer that you have high blood pressure?”; and “Have you ever been told by a doctor or assistant medical officer that you have high cholesterol?” For the analysis, respondents who answered “yes” to any of the conditions were categorized as having a long-term condition. For self-rated health, respondents were asked to answer using a five-point scale (excellent, good, fair, poor, very poor) to the question “How would you rate your health status?” The responses were then grouped into two categories: 1) low to moderate (comprising fair, poor or very poor responses); and 2) high (comprising excellent or good responses).

##### Caregiving-related characteristics

To determine living in the same household, the respondents were asked to answer “yes” or “no” to the question “In the last 12 months, from ... 2018 till today, did you provide care to your household member with long-term illness, elderly or disabled?” To determine if a caregiver had received training, the respondents were asked to answer “yes, by healthcare practitioner”, “yes, by other than healthcare practitioner” or “no” to the question “Were you trained to provide care to the care recipient?” Respondents who answered either “yes, by healthcare practitioner” or “yes, by other than healthcare practitioner” were coded as having received training. To determine if the caregiver had assistance, the respondents were asked to answer “yes” or “no” to the question “Who else provides care to the care recipient? 1) other family members, 2) domestic helper/maid, 3) nurse/other nursing professionals, 4) day-care/other institution, and 5) others such as neighbours”. Respondents who answered “yes” to any of the options were categorized as having assistance. Age of the care recipient was measured by the question “How old is (person receiving care)?” Age was initially measured as a continuous variable, in years, and then grouped into two categories: 1) 0–59 years, and 2) 60+ years. Duration of care was assessed by the question “How long have you been providing care to the care recipient?” The number of years was then grouped into two categories: 1) less than 2 years, and 2) two or more years.

#### Dependent variables

This study focused on the effect of caregiving roles on caregivers, with health, daily activities and social activities acting as the key outcome variables assessed in relation to the caregiving intensity level. The respondents were asked to answer “yes” or “no” to the following questions: “Have your role in providing care affected your health (physical and/or mental health)?’; “Have your role in providing care affected your daily, work or school activities?”; and “Have your role in providing care affected your social activities and others?” Hereinafter, daily, work or school activities will be referred to as daily activities.

### Statistical analysis

Secondary data analysis was conducted using STATA version 14 (Stata Corp, College Station, Texas, USA), and sample weights and study design were taken into consideration using a complex sampling design in all data analyses using the survey (svy) command. The weight used for estimation was based on the products of the inverse of the probability of sampling, non-response adjustment factor, and a post-stratification adjustment by age, gender, and ethnicity. Complex sample descriptive statistics were used to illustrate demographic, socioeconomic and other characteristics of the informal caregivers, according to caregiving intensity level. High intensity of care was defined as the provision of 20 or more hours of care per week, while low intensity of care was defined as the provision of 1–19 h of care per week [19, 22, 29]. Comparison of informal caregivers’ characteristics to level of intensity was performed using chi-square tests. Statistical significance was set at *p* <  0.05. Univariate analyses were performed separately for each level of intensity (high intensity and low intensity) to examine the association between independent variables (demographic, socioeconomic, health and caregiving-related characteristics), and dependent variables (health, daily and social activities), according to caregiving intensity level. Crude odds ratios (OR) were used to estimate strength of association between independent and dependent variables. Variables with a *p*-value of < 0.25 [30] in the univariate analysis were included in the final multivariate analysis models. Sex [23, 24, 31–33] and age [34–36] were also included in all final analysis models as these variables were found to be significantly associated with informal care in previous studies. For the final model, the enter variable selection method was used to determine the predictors after controlling for confounders. Adjusted OR with 95% confidence interval (CI) were calculated. The predictors were based on a *p*-value of < 0.05. The variance inflation factor (VIF) was used to test for multicollinearity, with a VIF of greater than 10 indicating a potential problem with multicollinearity [37]. The goodness of fit model was assessed using receiver operating characteristic curves and areas under the curve (AUC). An AUC of 0.9–1.0 was considered excellent, 0.8–0.9 very good, 0.7–0.8 good, 0.6–0.7 sufficient, 0.5–0.6 bad, and less than 0.5 not useful [38].

## Results

A total of 11,160 respondents, estimated to represent 21.3 million adults aged 18 years and over in Malaysia, were included in the analysis. Overall, 5.1% (95% CI = 4.45, 5.87) of adults in Malaysia reported providing informal care. Tables 1 and 2 summarise the demographic, socioeconomic, health and caregiving-related characteristics of caregivers stratified by care intensity level. Both low- and high-intensity caregivers had similar characteristics except for employment status and duration of care provision. High-intensity care was mostly provided by caregivers who were actively employed (63.0%) and had provided care for two or more years (69.2%).

**Table 1:** Demographic and socioeconomic characteristics of caregivers, stratified by care intensity level (*N* = 604)

Characteristic	Count, n (Unweighted)	Estimated population, N (Weighted)	Percentage (%)	Level of caregiving intensity				***p***-value
				1–19 h per week		20 and more hours per week		
				n	weighted %	n	weighted %	
**Sex**								
Male	232	412,091	37.8	154	37.0	78	38.9	0.722
Female	372	679,506	62.2	224	63.0	148	61.1	
**Ethnicity**								
Malay^a^	409	611,978	56.1	258	58.7	151	51.9	0.317
Non-Malay	195	479,619	43.9	120	41.3	75	48.1	
**Age (years)**								
18–34	133	357,802	32.8	87	30.5	46	36.3	0.424
35–59	318	519,676	47.6	198	50.9	120	42.5	
60+	153	214,119	19.6	93	18.6	60	21.2	
**Educational level:**								
No formal	32	70,346	6.4	23	7.4	9	5.0	0.422
Primary	135	188,625	17.3	76	14.3	59	21.9	
Secondary	309	579,614	53.1	199	53.7	110	52.2	
Tertiary	128	253,012	23.2	80	24.6	48	20.9	
**Marital status**								
Married	431	747,380	68.5	276	68.3	155	68.8	0.939
Not married (divorced, separated or never married)	173	344,217	31.5	102	31.7	71	31.2	
**Employment status:**								
Active	305	590,602	54.1	189	48.4	116	63.0	0.018
Inactive	299	500,995	45.9	189	51.6	110	37.0	
**Income**^**b**^								
Low	292	500,496	45.9	180	45.0	112	47.2	0.351
Middle	231	422,869	38.7	141	37.0	90	41.5	
High	81	168,232	15.4	57	18.0	24	11.3	
**Location**								
Urban	360	799,263	73.2	228	74.8	132	70.7	0.413
Rural	244	292,334	26.8	150	25.2	94	29.3	

^a^ Malay includes Orang Asli

^b^ Low income (comprised of those from quintile one and two), Middle income (comprised of those from quintile three and four), High income (comprised of those from the highest quintile)

**Table 2:** Health and caregiving-related characteristics of caregivers, stratified by care intensity level (*N* = 604)

Characteristic	Count, n (Unweighted)	Estimated population, N (Weighted)	Percentage (%)	Level of caregiving intensity				***p***-value
				1–19 h per week		20 and more hours per week		
				n	Weighted %	n	Weighted %	
***Health***								
**Self-reported health problems**								
Yes	202	361,073	33.1	128	35.2	74	29.8	0.339
No	402	730,523	66.9	250	64.8	152	70.2	
**Long-term condition**^**a**^								
Yes	209	325,627	29.8	134	31.4	75	27.4	0.447
No	395	765,970	70.2	244	68.6	151	72.6	
**Self-rated health**								
Excellent - Good	429	805,928	73.8	280	75.7	149	70.8	0.340
Fair - Very Poor	175	285,669	26.2	98	24.3	77	29.2	
***Caregiving-related***								
**Living in the same household:**								
Yes	525	933,687	85.5	324	82.7	201	89.9	0.103
No	79	157,910	14.5	54	17.3	25	10.1	
**Receive training**								
Yes	129	215,497	19.7	80	19.1	49	20.8	0.707
No	475	876,100	80.3	298	80.9	177	79.2	
**Has assistance**								
Yes	439	790,909	72.4	289	74.8	150	68.9	0.328
No	165	300,688	27.6	89	25.2	76	31.1	
**Duration of providing care (years)**								
Less than 2	378	664,631	60.9	142	45.0	77	30.8	0.014
2 and more	226	426,966	39.1	236	55.0	149	69.2	
***Caregiver outcomes***								
**Affect health**								
Yes	100	181,685	16.6	57	17.5	43	15.4	0.642
No	504	909,912	83.4	321	82.5	183	84.6	
**Affect daily activities**^**b**^								
Yes	142	289,245	26.5	75	25.3	67	28.4	0.613
No	462	802,351	73.5	303	74.7	159	71.6	
**Affect social activities**								
Yes	116	236,498	21.7	66	19.3	176	25.3	0.286
No	488	855,099	78.3	312	80.7	176	74.7	

^a^ Long-term condition refers to presence of any non-communicable diseases (diabetes, hypertension or hypercholesterolemia)

^b^ Daily activities refers to daily, work or school activities

Tables 3 and 4 display the results for impact on health, daily and social activities of caregivers providing low- and high-intensity care using univariate and multivariate logistic regression analyses, respectively.

**Table 3:** Univariate logistic regression model for impact on caregivers, stratified by caregiving intensity level

Factor	Low intensity level of care						High intensity level of care					
	Affect health		Affect daily activities^d^		Affect social activities		Affect health		Affect daily activities^d^		Affect social activities	
	Crude OR (95% CI)	***p***-value	Crude OR (95% CI)	***p***-value	Crude OR (95% CI)	***p***-value	Crude OR (95% CI)	***p***-value	Crude OR (95% CI)	p-value	Crude OR (95% CI)	***p***-value
***Demographic, socioeconomic and health***												
**Sex**												
Male	1.00 (ref)	–	1.00 (ref)	–	1.00 (ref)	–	1.00 (ref)	–	1.00 (ref)	–	1.00 (ref)	–
Female	4.28 (1.61–11.38)	0.004	2.20 (1.08–4.51)	0.031	2.25 (0.95–5.32)	0.065	2.49 (0.86–7.20)	0.092	1.15 (0.51–2.61)	0.733	1.76 (0.71–4.41)	0.224
**Ethnicity**												
Malay^a^	1.00 (ref)	–	1.00 (ref)	–	1.00 (ref)	–	1.00 (ref)	–	1.00 (ref)	–	1.00 (ref)	–
Non-Malay	1.48 (0.64–3.38)	0.356	3.12 (1.45–6.72)	0.004	1.65 (0.73–3.75)	0.232	0.71 (0.28–1.78)	0.461	1.47 (0.61–3.57)	0.391	2.30 (0.85–6.21)	0.100
**Age (years)**												
18–34	0.73 (0.18–3.01)	0.661	0.33 (0.08–1.26)	0.103	0.63 (0.20–1.94)	0.418	0.43 (0.13–1.47)	0.177	1.98 (0.63–6.17)	0.241	1.19 (0.37–3.84)	0.772
35–59	1.59 (0.64–3.97)	0.320	0.63 (0.19–2.01)	0.430	1.00 (0.40–2.48)	1.000	0.31 (0.12–0.80)	0.015	1.23 (0.52–2.89)	0.634	0.65 (0.26–1.65)	0.364
60+	1.00 (ref)	–	1.00 (ref)	–	1.00 (ref)	–	1.00 (ref)	–	1.00 (ref)	–	1.00 (ref)	–
**Educational level**												
No formal	1.00 (ref)	–	1.00 (ref)	–	1.00 (ref)	–	1.00 (ref)	–	1.00 (ref)	–	1.00 (ref)	–
Primary	0.83 (0.13–5.21)	0.845	2.57 (0.65–10.21)	0.180	0.95 (0.19–4.81)	0.952	1.25 (0.10–16.28)	0.864	1.14 (0.12–10.44)	0.908	1.42 (0.11–18.48)	0.790
Secondary	0.38 (0.08–1.74)	0.213	1.26 (0.35–4.57)	0.722	0.41 (0.11–1.54)	0.184	1.34 (0.12–15.30)	0.811	1.79 (0.21–15.27)	0.595	3.64 (0.31–42.30)	0.300
Tertiary	0.23 (0.04–1.43)	0.114	0.87 (0.18–4.19)	0.863	0.50 (0.11–2.28)	0.371	2.96 (0.24–36.53)	0.396	1.32 (0.14–12.18)	0.807	3.97 (0.31–50.20)	0.286
**Marital status**												
Married	2.82 (0.94–8.44)	0.064	0.84 (0.30–2.31)	0.732	1.32 (0.59–2.94)	0.500	0.51 (0.20–1.32)	0.163	0.84 (0.37–1.89)	0.666	0.69 (0.28–1.66)	0.405
Not married (divorced, separated or never married)	1.00 (ref)	–	1.00 (ref)	–	1.00 (ref)	–	1.00 (ref)	–	1.00 (ref)	–	1.00 (ref)	–
**Employment status**												
Active	1.00 (ref)	–	1.00 (ref)	–	1.00 (ref)	–	1.00 (ref)	–	1.00 (ref)	–	1.00 (ref)	–
Inactive	1.41 (0.60–3.28)	0.427	2.10 (0.88–5.03)	0.096	2.05 (0.93–4.49)	0.074	1.40 (0.58–3.40)	0.457	0.78 (0.35–1.78)	0.561	0.86 (0.37–1.99)	0.718
**Income**^**b**^												
Low	2.28 (0.60–8.70)	0.228	1.45 (0.35–6.06)	0.613	1.62 (0.49–5.40)	0.427	0.79 (0.16–4.00)	0.778	1.13 (0.26–4.92)	0.867	1.05 (0.29–3.82)	0.938
Middle	0.74 (0.19–2.86)	0.667	1.41 (0.33–6.07)	0.644	0.89 (0.27–2.97)	0.853	0.79 (0.16–3.79)	0.766	1.32 (0.33–5.25)	0.688	0.69 (0.19–2.50)	0.576
High	1.00 (ref)	–	1.00 (ref)	–	1.00 (ref)	–	1.00 (ref)	–	1.00 (ref)	–	1.00 (ref)	–
**Location**												
Urban	1.00 (ref)	–	1.00 (ref)	–	1.00 (ref)	–	1.00 (ref)	–	1.00 (ref)	–	1.00 (ref)	–
Rural	1.02 (0.46–2.26)	0.964	0.82 (0.39–1.74)	0.611	0.80 (0.36–1.78)	0.592	0.74 (0.26–2.12)	0.574	0.65 (0.29–1.50)	0.316	0.36 (0.14–0.88)	0.026
**Self-reported health problems**												
Yes	1.54 (0.65–3.63)	0.324	1.29 (0.48–3.44)	0.611	1.14 (0.52–2.51)	0.742	1.96 (0.78–4.88)	0.150	1.03 (0.42–2.54)	0.950	0.78 (0.29–2.07)	0.618
No	1.00 (ref)	–	1.00 (ref)	–	1.00 (ref)	–	1.00 (ref)	–	1.00 (ref)	–	1.00 (ref)	–
**Long-term condition**^**c**^												
Yes	2.52 (1.08–5.87)	0.032	1.36 (0.59–3.13)	0.466	1.43 (0.63–3.29)	0.394	1.98 (0.79–4.98)	0.145	1.10 (0.47–2.55)	0.828	0.77 (0.30–1.96)	0.587
No	1.00 (ref)	–	1.00 (ref)	–	1.00 (ref)	–	1.00 (ref)	–	1.00 (ref)	–	1.00 (ref)	–
**Self-rated health**												
Excellent - Good	1.00 (ref)	–	1.00 (ref)	–	1.00 (ref)	–	1.00 (ref)	–	1.00 (ref)	–	1.00 (ref)	–
Fair - Very Poor	1.36 (0.52–3.54)	0.525	1.13 (0.49–2.58)	0.778	1.80 (0.74–4.33)	0.192	2.28 (0.92–5.66)	0.074	1.13 (0.48–2.67)	0.779	0.90 (0.34–2.37)	0.834
***Caregiving-related***												
**Living in the same household**												
Yes	1.09 (0.25–4.76)	0.905	0.67 (0.25–1.78)	0.416	0.65 (0.20–2.04)	0.457	0.80 (0.21–3.03)	0.739	1.52 (0.44–5.27)	0.511	7.32 (1.19–45.22)	0.032
No	1.00 (ref)	–	1.00 (ref)	–	1.00 (ref)	–	1.00 (ref)	–	1.00 (ref)	–	1.00 (ref)	–
**Receive training**												
Yes	1.55 (0.59–4.08)	0.370	1.60 (0.56–4.56)	0.381	1.26 (0.51–3.14)	0.613	3.12 (1.27–7.64)	0.013	1.80 (0.69–4.65)	0.227	3.16 (1.17–8.54)	0.024
No	1.00 (ref)	–	1.00 (ref)	–	1.00 (ref)	–	1.00 (ref)	–	1.00 (ref)	–	1.00 (ref)	–
**Has assistance**												
Yes	1.00 (ref)	–	1.00 (ref)	–	1.00 (ref)	–	1.00 (ref)	–	1.00 (ref)	–	1.00 (ref)	–
No	1.70 (0.64–4.55)	0.286	2.20 (0.78–6.20)	0.135	1.35 (0.51–3.57)	0.538	2.78 (1.06–7.26)	0.037	2.65 (1.04–6.78)	0.042	2.41 (0.88–6.62)	0.088
**Duration of providing care (years)**												
Less than 2	1.00 (ref)	–	1.00 (ref)	–	1.00 (ref)	–	1.00 (ref)	–	1.00 (ref)	–	1.00 (ref)	–
2 and more	2.92 (1.37–6.24)	0.006	0.85 (0.36–2.02)	0.711	1.23 (0.57–2.67)	0.603	0.66 (0.27–1.63)	0.369	1.46 (0.62–3.40)	0.378	2.89 (1.16–7.18)	0.023

*CI* Confidence Interval, *OR* Odds Ratio, *ref.* reference category

^a^ Malay includes Orang Asli

^b^ Low income (comprised of those from quintile one and two), Middle income (comprised of those from quintile three and four), High income (comprised of those from the highest quintile)

^c^ Long-term condition refers to presence of any non-communicable diseases (diabetes, hypertension or hypercholesterolemia)

^d^ Daily activities refers to daily, work or school activities

**Table 4:** Multivariate logistic regression model for impact on caregivers, stratified by caregiving intensity level

Factor	Low intensity level of care						High intensity level of care					
	Affect health		Affect daily activities^d^		Affect social activities		Affect health		Affect daily activities^d^		Affect social activities	
	Adjusted OR (95% CI)	p-value	Adjusted OR (95% CI)	p-value	Adjusted OR (95% CI)	p-value	Adjusted OR (95% CI)	p-value	Adjusted OR (95% CI)	p-value	Adjusted OR (95% CI)	p-value
***Demographic, socioeconomic and health***												
**Sex**												
Male	1.00 (ref)	–	1.00 (ref)	–	1.00 (ref)	–	1.00 (ref)	–	1.00 (ref)	–	1.00 (ref)	–
Female	**3.33 (1.26–8.75)**	**0.015**	1.56 (0.76–3.22)	0.227	1.67 (0.66–4.22)	0.281	3.15 (0.89–11.18)	0.076	0.94 (0.45–1.95)	0.858	1.78 (0.63–5.04)	0.275
**Ethnicity**												
Malay^a^			1.00 (ref)	–	1.00 (ref)	–					1.00 (ref)	–
Non-Malay			**2.91 (1.40–6.08)**	**0.004**	1.49 (0.65–3.39)	0.344					**3.34 (1.25–8.93)**	**0.016**
**Age (years)**												
18–34	3.54 (0.65–19.33)	0.144	0.48 (0.15–1.56)	0.222	0.88 (0.27–2.94)	0.839	0.32 (0.08–1.36)	0.122	2.31 (0.71–7.47)	0.163	0.68 (0.19–2.44)	0.553
35–59	**3.75 (1.22–11.51)**	**0.021**	0.86 (0.32–2.33)	0.770	1.31 (0.48–3.57)	0.596	**0.20 (0.05–0.80)**	**0.023**	1.22 (0.47–3.15)	0.681	0.30 (0.08–1.18)	0.086
60+	1.00 (ref)	–	1.00 (ref)	–	1.00 (ref)	–	1.00 (ref)	–	1.00 (ref)	–	1.00 (ref)	–
**Educational level**												
No formal	1.00 (ref)	–									1.00 (ref)	–
Primary	1.22 (0.22–6.91)	0.822									1.05 (0.10–10.52)	0.968
Secondary	0.65 (0.13–3.11)	0.584									3.86 (0.35–42.12)	0.267
Tertiary	0.42 (0.06–3.18)	0.401									9.00 (0.68–119.47)	0.096
**Marital status**												
Married	2.57 (0.90–7.29)	0.077					0.38 (0.13–1.14)	0.084				
Not married (divorced, separated or never married)	1.00 (ref)	–					1.00 (ref)	–				
**Employment status**												
Active			1.00 (ref)	–	1.00 (ref)	–						
Inactive			1.32 (0.56–3.11)	0.521	1.62 (0.68–3.87)	0.274						
**Income**^**b**^												
Low	1.79 (0.50–6.42)	0.372										
Middle	0.62 (0.20–1.93)	0.412										
High	1.00 (ref)	–										
**Location**												
Urban											1.00 (ref)	–
Rural											0.57 (0.19–1.71)	0.319
**Self-reported health problems**												
Yes							2.58 (0.76–8.75)	0.129				
No							1.00 (ref)	–				
**Long-term condition**^**c**^												
Yes	**3.43 (1.42–8.32)**	**0.006**					0.79 (0.18–3.45)	0.758				
No	1.00 (ref)	–					1.00 (ref)	–				
**Self-rated health**												
Excellent - Good					1.00 (ref)	–	1.00 (ref)	–				
Fair - Very Poor					1.65 (0.63–4.33)	0.312	1.27 (0.43–3.70)	0.664				
***Caregiving-related***												
**Living in the same household**												
Yes											9.34 (0.89–97.64)	0.062
No											1.00 (ref)	–
**Receive training**												
Yes							**4.50 (1.61–12.56)**	**0.004**	1.88 (0.74–4.77)	0.184	**4.46 (1.82–10.96)**	**0.001**
No							1.00 (ref)	–	1.00 (ref)	–	1.00 (ref)	–
**Has assistance**												
Yes			1.00 (ref)	–			1.00 (ref)	–	1.00 (ref)	–	1.00 (ref)	–
No			1.61 (0.66–3.95)	0.299			**3.18 (1.07–9.45)**	**0.037**	**2.96 (1.16–7.54)**	**0.023**	1.67 (0.63–4.43)	0.303
**Duration of providing care (years)**												
Less than 2	1.00 (ref)	–									1.00 (ref)	–
2 and more	2.02 (0.92–4.43)	0.080									**3.73 (1.12–12.42)**	**0.032**

Statistically significant *p*-values (< 0.05) are shown in bold. Multicollinearity was unlikely. Overall fit of the model for each binary logit was checked using weighted area under receiver operating characteristic (ROC) curve [model affect health (low intensity), 0.803; model affect daily activities (low intensity), 0.707; model affect social activities (low intensity), 0.658; model affect health (high intensity), 0.799; model affect daily activities (high intensity), 0.654; model affect social activities (high intensity), 0.819]. Models were considered fit based on the area under the curve

*CI* Confidence Interval, *OR* Odds Ratio, *ref.* reference category

^a^ Malay includes Orang Asli

^b^ Low income (comprised of those from quintile one and two), Middle income (comprised of those from quintile three and four), High income (comprised of those from the highest quintile)

^c^ Long-term condition refers to presence of any non-communicable diseases (diabetes, hypertension or hypercholesterolemia)

^d^ Daily activities refers to daily, work or school activities

### Impact on health, daily and social activities of low-intensity caregivers

In the univariate analysis, variables that had a significant association with impact on the health of caregivers providing a low-intensity level of care were sex (*p* = 0.004), long-term conditions (*p* = 0.032), and duration of care (*p* = 0.006). Variables that had a significant association with impact on daily activities of caregivers providing a low-intensity level of care were sex (*p* = 0.031) and ethnicity (p = 0.004). None of the variables had a significant association with impact on social activities of caregivers providing a low-intensity level of care.

After controlling for all other variables in the model, the likelihood of impact on the health of caregivers providing a low-intensity level of care was higher among females (OR = 3.33, 95% CI = 1.26, 8.75). Those aged 35–59 years were 3.75 times (95% CI = 1.22, 11.51) more likely to have an impact on health compared to those aged 60 and above. Those with long-term conditions (OR = 3.43, 95% CI = 1.42, 8.32) had a higher likelihood of having an impact on health compared to those without. In terms of impact on daily activities, non-Malays (OR = 2.91, 95% CI = 1.40, 6.08) were more likely to be affected than Malays. No variable was found to be significant for impact on social activities of caregivers providing a low-intensity level of care.

### Impact on health, daily and social activities of high-intensity caregivers

In the univariate analysis, variables that had a significant association with impact on the health of caregivers providing a high-intensity level of care were received training (*p* = 0.013) and had assistance (*p* = 0.037). The only variable that had a significant association with impact on daily activities of caregivers providing high-intensity care was had assistance (*p* = 0.042). Variables that had a significant association with impact on social activities of caregivers providing high-intensity care were location (*p* = 0.026), living in the same household (*p* = 0.032), received training (*p* = 0.024), and duration of care (*p* = 0.023).

After controlling for all other variables in the model, the likelihood of impact on the health of caregivers providing high-intensity care was lower among those aged 35–59 years (OR = 0.20, 95% CI = 0.05, 0.80) compared to those aged 60 and above. Those who received training were 4.50 times (95% CI = 1.61, 12.56) more likely to have an impact on health compared to those without training. Those without assistance (OR = 3.18, 95% CI = 1.07, 9.45) had a higher likelihood of having an impact on health compared to those with assistance. In terms of impact on daily activities, those without assistance (OR = 2.96, 95% CI = 1.16, 7.54) had a higher likelihood of having an impact on daily activities compared to those with assistance. In terms of impact on social activities of caregivers providing high-intensity care, non-Malays (OR = 3.34, 95% CI = 1.25, 8.93) were more likely to be affected than Malays. Those who received training were 4.46 times (95% CI = 1.82, 10.96) more likely to have an impact on social activities compared to those without training. High-intensity caregivers providing care for two or more years (OR = 3.73, 95% CI = 1.12, 12.42) were more likely to be affected in terms of social activities compared to those who provided care for less than 2 years.

Multicollinearity analysis revealed that all variables had VIFs of less than 5 (ranging from 1.04 to 1.50), indicating that multicollinearity was unlikely. Models were considered fit based on the AUCs of more than 0.6 for each model.

## Discussion

This study investigated the predictive factors associated with the effects of caregiving roles on caregivers in relation to intensity of care. Our findings revealed that the effects of caregiving on health, daily and social activities of informal caregivers differed based on predictive factors and level of caregiving intensity.

We found that high-intensity caregivers were primarily actively employed and provided care for two or more years. Previous studies [9, 21, 22, 39] have shown that intensive caregiving affected employment or interfered with work, leading to reduced working hours, absenteeism, decreased job performance, or early retirement. Our study did not assess job interference, which warrants further exploration. A nationwide panel survey conducted in Japan reported that caregiving persisted among non-working caregivers who performed high-intensity caregiving for a year [19] and published studies have found that caregiving intensity corresponds with the magnitude of health effects [13, 18]. Hence, further exploration is warranted into the effects of caregiving intensity on caregivers.

Published studies reported that caregiver demographics such as sex and age were associated with negative impacts on caregivers [13, 40, 41]. Our study found that, among low-intensity caregivers, women’s health was more likely to be adversely affected by caregiving than men’s. In terms of age, the health of middle-aged adults was more likely to be adversely affected by caregiving than adults aged 60 and older in both low- and high-intensity caregivers. According to a meta-analysis of findings from 229 studies, female caregivers reported higher levels of caregiver burden and distress, and lower levels of physical health and subjective well-being, than male caregivers [42]. Gender disparities in health among the caregivers might be explained by the fact that women spend more hours performing caregiving and partake in more caregiving tasks [6, 42]. Additionally, female and middle-aged caregivers may have the complicating factors of responsibility for their own children and careers, while still providing informal care. Multiple and potentially overlapping roles may result in additional burden [43] resulting from difficulty balancing caregiving with other family and employment responsibilities [3, 44], which may subsequently cause detrimental effects on health [3].

Malaysia is a multiracial nation with varied cultures, with Malays forming the largest ethnic group in the country with 69.6% of Malaysia’s population, followed by Chinese, Indians and others [45]. This study found that ethnicity was associated with negative consequences on low-intensity caregivers’ daily activities and on high-intensity caregivers’ social activities, with non-Malay caregivers more affected than Malay caregivers. The results of this study are consistent with local studies conducted among dementia caregivers in Malaysia, which reported that Chinese caregivers had higher levels of burden than Malay caregivers [46, 47]. Many studies have also found that filial obligation and ethnocentric societal norms were components of socio-cultural differences that influenced the caregiving process [4, 5, 47–53]. According to Malay culture and Islam, God’s will is above all, and because difficulties are seen as God’s will, Muslims should accept them. Acceptance, faith and religious practices were found to be common and effective coping strategies used by caregivers in Malaysia [54].

Our study showed that low-intensity caregivers with long-term conditions were more likely to suffer from health impacts. A previous study found that caregivers often neglect their own healthcare needs due to the caregiving process, which may take a toll and lead to worsening of pre-existing conditions [55]. Since the caregiver role is often associated with physical and psychiatric diseases [10, 56], it serves as a powerful predictor of greater risk for psychological distress, anxiety, depression and cardiovascular reactivity [12, 56, 57]. However, while the caregiving role can have a negative impact on caregivers, research has shown that the effects can be mitigated by assessing [58] and addressing caregiver needs [59, 60].

Our study found that caregivers providing high-intensity care for two or more years were more likely to suffer social strains compared to caregivers who provided high-intensity care for less than 2 years. Multiple years of filling a caregiving role have been associated with a greater burden among caregivers [61]. Provision of high-intensity care likely requires a high level of commitment; when coupled with a longer care duration, caregiving duties might affect availability to participate in social activities [3]. On the other hand, another study reported a significantly greater burden for short-duration caregiving and a lower burden for long-duration caregiving [62], which was most likely due to the development of coping skills and mechanisms that made caregiving easier over time [63, 64].

Rather surprisingly, the current study found that the health and social activities of trained caregivers who provided high-intensity care were more likely to be affected than caregivers who had not been trained. One may postulate that, although training facilitates the provision of quality care by promoting knowledge and care techniques for caregivers, it does not explicitly translate to caregivers’ health and social activities. Caregivers require both knowledge and skill to provide care, and training caregivers has been demonstrated to be beneficial in the successful carrying out of their responsibilities [65]. As such, in Malaysia, the provision of training for caregivers is outlined in the National Policy for Older Persons and Plan of Action for the Older Persons [66, 67]. However, published articles have pointed out that most interventions have been shown to have domain-specific outcomes. For example, mindfulness-based interventions have a positive influence on reducing depression, and occupational therapy training have a direct impact on strengthening caregivers’ competence and confidence [68]. As caregivers have vastly different needs, interventions must be tailored according to the specific needs of the individual caregiver.

Demographic and socioeconomic characteristics, as well as other factors, can moderate the effects of the other variables on caregivers’ health, daily activities and social activities. Previous studies found that coping and social support moderate the negative effects of the caregiving role, as it acts as a buffer to mitigate the impact of care stressors on the caregiver [69–71]. The effect of individual-level risk factors on caregiver outcomes has been found to be moderated by neighbourhood characteristics [72] and a recently published paper has concluded that the effect of caregiving time on depressive symptoms is moderated by employment status and gender [73]. However, as the moderating effect of demographic, socioeconomic and other factors were not addressed in the current study, there is a possibility that these factors may mask the effects of caregiving on caregivers’ health, daily activities and social activities.

Support for informal caregivers utilizing services and expertise for the provision of integrated social care is insufficient in both the public and private sectors in Malaysia [4, 5]. Our study showed that the health and daily activities of caregivers without assistance who provided high-intensity care were more likely to be affected than caregivers who received assistance. Studies have expressed the need for and relevance of establishing a strong informal care support system to meet caregivers’ needs in helping them cope with caregiving tasks and responsibilities [4, 8]. The European Union has implemented a policy that provides financial support, counselling, respite care and training to support informal care [7], and England has awarded caregivers legal recognition to boost motivation to continue caregiving responsibilities [74]. England, France and the Netherlands have also implemented policies to stimulate caregivers’ physical and mental well-being through the provision of specific support services, including training/education, respite care and counselling [7, 74]. The Central Information and Communication Centres in France provide support individually, meeting with caregivers daily and connecting them with medical professionals. The Netherlands uses a preventive counselling and support approach, in which skilled social workers provide caregivers with information and follow-up telephone interviews on a three-month basis to prevent the risk of caregivers developing mental health issues [75]. The presence of an established and systematic caregiver support system and network can shorten the required hours of care and reduce caregiver burden. In Malaysia, the support services most sought after by caregivers were home help services and home nursing services [5].

With a population that is ageing with an increased prevalence of comorbidities and disability, demand for long-term care in Malaysia is increasing as well. Informal care is considered an important component and valuable resource for long-term health and social care [76]. A concerted effort is required to support the well-being of informal caregivers to ensure that the formal long-term care system is continuously complemented [15, 76]. The elderly are the primary recipients of informal care in Malaysia [26], and the current structure for long-term care is dependent on informal care to support the needs of the elderly [5, 77]. Despite the government’s efforts and initiatives to support informal caregivers by the provision of assistance for care, household chores, treatment and respite care through the National Policy for Older Persons and Plan of Action for the Older Persons [66, 67], informal caregivers in Malaysia reported that they mainly received support from family members, with limited support from professional, day or institutional care systems [26]. This may be due to limited availability of these services [77] despite the government offering institutional- and home-care support for long-term care recipients.

Our study had several limitations. As NHMS was a cross-sectional study, causal relationships between caregiving intensity and impact on health, daily and social activities could not be determined. Another limitation to consider is the lack of depth in the topic as the questions from the NHMS were general in nature. Other variables such as the severity of the care recipient’s health condition, use of respite care and presence of any short breaks from caregiving roles were not included in the study, which deters possible associations with the studied predictors. This suggests that more detailed research that includes these variables and future exploration into the causal relationships of informal care and caregiving intensity are justified. Additionally, no previous research was conducted in a local context pertaining to the effects of caregiving intensity, which made comparison impossible. However, this study provides relevant evidence on the current situation of informal caregivers in Malaysia.

Despite these limitations, the main strength of this study includes its large sample size, which involved a nationwide population that enabled the generalisation of results for Malaysia. As this was the first national study on informal caregivers conducted in Malaysia, the findings from this study could help enrich discussion pertaining to informal care and guide future programmes, policies and potential research.

## Conclusion

Our study indicates that both low- and high-intensity caregivers have common features, with the exception of employment status and care duration. Caregiving, regardless of intensity, has a significant impact on caregivers. In order to reduce the negative consequences of caregiving responsibilities, all caregivers need assistance from the community and government, including but not limited to information to aid understanding, skills to better carry out caregiving roles, respite to enable participation in other activities, and financial support that is customised to their needs. Informal caregivers are valuable resources in the provision of long-term health and social care. As such, by addressing the factors contributing to the negative effects of caregiving, a continuation of informal caregiving can be sustained through policies supporting the growing demand for informal care necessitated by an ageing population and higher life expectancy in Malaysia.

## Data Availability

The dataset that supports the findings of this article is not publicly available to protect participant privacy. Request for data can be obtained from the Head of Centre for Biostatistics & Data Repository, National Institutes of Health, Ministry of Health Malaysia on reasonable request and with the permission from the Director General of Health, Malaysia.

## Abbreviations

NHMS: National Health and Morbidity Survey
DOSM: Department of Statistics Malaysia
EBs: Enumeration Blocks
LQs: Living Quarters
MREC: Medical Research and Ethics Committee
NMRR: National Medical Research Register
NCD: Non-communicable disease
OR: Odds Ratio
CI: Confidence Interval
‘svy’: Survey command in STATA
VIF: Variance inflation factor
AUC: Areas under the curve

## References

[1] Colombo F, Mercier J (2012). Help wanted? Fair and sustainable financing of long-term care services. Appl Econ Perspect Policy.

[2] Broese van Groenou MI, De Boer A (2016). Providing informal care in a changing society. Eur J Ageing.

[3] Committee on Family Caregiving for Older Adults, Board on Health Care Services, Health and Medicine Division, National Academies of Sciences, Engineering and M (2016). Families caring for an aging America.

[4] Abu Bakar SH, Weatherley R, Omar N, Abdullah F, Mohamad Aun NS (2014). Projecting social support needs of informal caregivers in Malaysia. Heal Soc Care Community.

[5] Goh ZY, Lai MM, Lau SH, Ahmad N (2013). The formal and informal long-term caregiving for the elderly: the Malaysian experience. Asian Soc Sci.

[6] Schulz R, Beach SR, Czaja SJ, Martire LM, Monin JK (2020). Family caregiving for older adults. Annu Rev Psychol.

[7] Courtin E, Jemiai N, Mossialos E (2014). Mapping support policies for informal carers across the European Union. Health Policy.

[8] Plöthner M, Schmidt K, De Jong L, Zeidler J, Damm K (2019). Needs and preferences of informal caregivers regarding outpatient care for the elderly: a systematic literature review. BMC Geriatr.

[9] Stanfors M, Jacobs JC, Neilson J (2019). Caregiving time costs and trade-offs: gender differences in Sweden, the UK, and Canada. SSM - Popul Heal.

[10] Bauer JM, Sousa-Poza A (2015). Impacts of informal caregiving on caregiver employment, health, and family. J Popul Ageing.

[11] O’Reilly D, Rosato M, Maguire A, Wright D (2015). Caregiving reduces mortality risk for most caregivers: a census-based record linkage study. Int J Epidemiol.

[12] Pinquart M, Sörensen S (2007). Correlates of physical health of informal caregivers: a meta-analysis. J Gerontol Ser B Psychol Sci Soc Sci.

[13] Schulz R, Sherwood PR (2008). Physical and mental health effects of family caregiving. Am J Nurs.

[14] Deeken JF, Taylor KL, Mangan P, Yabroff KR, Ingham JM (2003). Care for the caregivers: a review of self-report instruments developed to measure the burden, needs, and quality of life of informal caregivers. J Pain Symptom Manag.

[15] Lopez-Hartmann M, Wens J, Verhoeven V, Remmen R (2012). The effect of caregiver support interventions for informal caregivers of community-dwelling frail elderly: a systematic review. Int J Integr Care.

[16] Do YK, Norton EC, Stearns SC, Van Houtven CH (2015). Informal care and caregiver’s health. Health Econ.

[17] Buyck JF, Bonnaud S, Boumendil A, Andrieu S, Bonenfant S, Goldberg M, Zins M, Ankri J (2011). Informal caregiving and self-reported mental and physical health: results from the gazel cohort study. Am J Public Health.

[18] Bremer P, Cabrera E, Leino-Kilpi H, Lethin C, Saks K, Sutcliffe C, Soto M, Zwakhalen SM, Wübker A, RightTimePlaceCare Consortium (2015). Informal dementia care: consequences for caregivers’ health and health care use in 8 European countries. Health Policy.

[19] Kumagai N (2017). Distinct impacts of high intensity caregiving on caregivers’ mental health and continuation of caregiving. Heal Econ Rev.

[20] Ueshima H, Yozu A, Takahashi H, Noguchi H, Tamiya N (2020). The association between activities of daily living and long hours of care provided by informal caregivers using a nationally representative survey in Japan. SSM Popul Heal.

[21] Jacobs JC, Laporte A, Van Houtven CH, Coyte PC (2014). Caregiving intensity and retirement status in Canada. Soc Sci Med.

[22] Jacobs JC, Van Houtven CH, Tanielian T, Ramchand R (2019). Economic spillover effects of intensive unpaid caregiving. Pharmacoeconomics..

[23] Oliva-Moreno J, Peña-Longobardo LM, García-Mochón L, Del Río Lozano M, Metcalfe IM, Del Mar García-Calvente M (2019). The economic value of time of informal care and its determinants (the CUIDARSE study). PLoS One.

[24] Bom J, Bakx P, Schut F, Van Doorslaer E (2019). The impact of informal caregiving for older adults on the health of various types of caregivers: a systematic review. Gerontologist..

[25] Hirst M (2005). Carer distress: a prospective, population-based study. Soc Sci Med.

[26] Institute for Health Systems Research & Institute for Public Health (2020). National Health and Morbidity Survey (NHMS) 2019: Vol II: Healthcare Demand.

[27] Institute for Health Systems Research (2018). Revision of healthcare demand questionnaire for national health and morbidity survey (NHMS) 2019.

[28] Chong DWQ, Jawahir S, Tan EH, Sararaks S. Redesigning a healthcare demand questionnaire for national population survey: experience of a developing country. Int J Environ Res Public Health. 2021;18(9):4435. 10.3390/ijerph18094435. 10.3390/ijerph18094435 PMC8122655 33921985

[29] Ramsay S, Grundy E, O’Reilly D (2013). The relationship between informal caregiving and mortality: an analysis using the ONS longitudinal study of England and Wales. J Epidemiol Community Health.

[30] Bursac Z, Gauss CH, Williams DK, Hosmer DW (2008). Purposeful selection of variables in logistic regression. Source Code Biol Med.

[31] Brazil K, Thabane L, Foster G, Bédard M (2009). Gender differences among Canadian spousal caregivers at the end of life. Heal Soc Care Community.

[32] Kim Y, Baker F, Spillers RL (2007). Cancer caregivers’ quality of life: effects of gender, relationship, and appraisal. J Pain Symptom Manag.

[33] Yee JL, Schulz R (2000). Gender differences in psychiatric morbidity among family caregivers: a review and analysis. Gerontologist..

[34] Anderson LA, Edwards VJ, Pearson WS, Talley RC, McGuire LC, Andresen EM (2013). Adult caregivers in the United States: characteristics and differences in well-being, by caregiver age and caregiving status. Prev Chronic Dis.

[35] Cook SK, Snellings L, Cohen SA (2018). Socioeconomic and demographic factors modify observed relationship between caregiving intensity and three dimensions of quality of life in informal adult children caregivers. Health Qual Life Outcomes.

[36] Yakubu YA, Schutte DW (2018). Caregiver attributes and socio-demographic determinants of caregiving burden in selected low-income communities in cape town, South Africa. J Compassionate Heal Care.

[37] Hair JF, Black WC, Babin BJ, Anderson RE. Dependence techniques – Metric outcomes. In: Multivariate Data Analysis. 8th ed. Hampshire: Cengage Learning EMEA; 2019. p. 316.

[38] Šimundić A-M (2009). Measures of diagnostic accuracy: basic definitions. EJIFCC..

[39] Carr E, Murray ET, Zaninotto P, Cadar D, Head J, Stansfeld S (2018). The association between informal caregiving and exit from employment among older workers: prospective findings from the UK household longitudinal study. J Gerontol Ser B Psychol Sci Soc Sci.

[40] Vellone E, Fida R, Cocchieri A, Sili A, Piras G, Alvaro R (2011). Positive and negative impact of caregiving to older adults: a structural equation model. Prof Inferm.

[41] Meiland FJM, Danse JAC, Wendte JF, Klazinga NS, Gunning-Schepers LJ (2001). Caring for relatives with dementia — caregiver experiences of relatives of patients on the waiting list for admission to a psychogeriatric nursing home in the Netherlands. Scand J Public Health.

[42] Pinquart M, Sörensen S (2006). Gender differences in caregiver stressors, social resources, and health: an updated meta-analysis. J Gerontol Ser B Psychol Sci Soc Sci.

[43] de Almeida Mello J, Macq J, Van Durme T, Cès S, Spruytte N, Van Audenhove C (2017). The determinants of informal caregivers’ burden in the care of frail older persons: a dynamic and role-related perspective. Aging Ment Health.

[44] Stephens MAP, Townsend AL, Martire LM, Druley JA (2001). Balancing parent care with other roles: Interrole conflict of adult daughter caregivers. J Gerontol Ser B Psychol Sci Soc Sci.

[45] Department of Statistics Malaysia (2020). Current population estimates, Malaysia, 2020.

[46] Aman Z, Liew SM, Ramdzan SN, Philp I, Khoo EM (2020). The impact of caregiving on caregivers of older persons and its associated factors: a cross-sectional study. Am J Phys Regul Integr Comp Phys.

[47] Choo WY, Low WY, Karina R, Poi PJH, Ebenezer E, Prince MJ (2003). Social support and burden among caregivers of patients with dementia in Malaysia. Asia-Pacific J Public Heal.

[48] Connell CM, Janevic MR, Gallant MP (2001). The costs of caring: impact of dementia on family caregivers. J Geriatr Psychiatry Neurol.

[49] Covinsky KE, Newcomer R, Fox P, Wood J, Sands L, Dane K, Yaffe K (2003). Patient and caregiver characteristics associated with depression in caregivers of patients with dementia. J Gen Intern Med.

[50] Haley WE, Levine EG, Brown SL, Bartolucci AA (1987). Stress, appraisal, coping, and social support as predictors of adaptational outcome among dementia caregivers. Psychol Aging.

[51] Lim YM, Luna I, Cromwell SL, Phillips LR, Russell CK, Torres De Ardon E (1996). Toward a cross-cultural understanding of family caregiving burden. West J Nurs Res.

[52] McConaghy R, Caltabiano ML (2005). Caring for a person wih dementia: exploring relationships between perceived burden, depression, coping and well-being. Nurs Health Sci.

[53] Salleh MR (1994). The burden of care of schizophrenia in Malay families. Acta Psychiatr Scand.

[54] Azman A, Jamir Singh PS, Sulaiman J (2017). Caregiver coping with the mentally ill: a qualitative study. J Ment Health.

[55] Faronbi JO, Faronbi GO, Ayamolowo SJ, Olaogun AA (2019). Caring for the seniors with chronic illness: the lived experience of caregivers of older adults. Arch Gerontol Geriatr.

[56] Zegwaard MI, Aartsen MJ, Cuijpers P, Grypdonck MH (2011). Review: a conceptual model of perceived burden of informal caregivers for older persons with a severe functional psychiatric syndrome and concomitant problematic behaviour. J Clin Nurs.

[57] Schulz R, O’Brien AT, Bookwala J, Fleissner K (1995). Psychiatric and physical morbidity effects of dementia caregiving: prevalence, correlates, and causes. Gerontologist..

[58] Feinberg L, Arl H (2012). Assessing family caregiver needs: policy and practice considerations.

[59] Kaye LW, Turner W, Butler SS, Downey R, Cotton A (2003). Early intervention screening for family caregivers of older relatives in primary care practices:establishing a community health service alliance in rural America. Fam Community Heal.

[60] Reinhard SC, Given B, Petlick NH, Bemis A (2008). Supporting family caregivers in providing care.

[61] Özmen S, Yurttas A. Determination of care burden of caregivers of patients with multiple sclerosis in Turkey. Behav Neurol. 2018;2018:7205046. 10.1155/2018/7205046. 10.1155/2018/7205046 PMC5884278 29755612

[62] Zainuddin J, Arokiasamy JT, Poi P (2003). Caregiving burden is associated with short rather than long duration of care for older persons. Asia-Pacific J Public Heal.

[63] Johnson PD (1998). Rural stroke caregivers: a qualitative study of the positive and negative response to the caregiver role. Top Stroke Rehabil.

[64] Seltzer MM, Li LW (1996). The transitions of caregiving: subjective and objective definitions. Gerontologist..

[65] Powers SE (2006). The family caregiver program: design and effectiveness of an education intervention. Home Healthc Nurse.

[66] Plan of Action for the Older Persons. Ministry of Women, Family and Community Development Malaysia; 1999.

[67] National Plan of Action for Health Care of Older Person (1997). Ministry of Health Malaysia.

[68] Cheng ST, Zhang F (2020). A comprehensive meta-review of systematic reviews and meta-analyses on nonpharmacological interventions for informal dementia caregivers. BMC Geriatr.

[69] Chang HJ (2009). The correlation of home care with family caregiver burden and depressive mood: an examination of moderating functions. Int J Gerontol.

[70] Haya MAN, Ichikawa S, Wakabayashi H, Takemura Y. Family caregivers’ perspectives for the effect of social support on their care burden and quality of life: A mixed-method study in rural and sub-urban central Japan. Tohoku J Exp Med. 2019;247(3):197–207. 10.1620/tjem.247.197 30890666

[71] Saffari M, Koenig HG, O’Garo KN, Pakpour AH. Mediating effect of spiritual coping strategies and family stigma stress on caregiving burden and mental health in caregivers of persons with dementia. London: Dementia; 2018:1471301218798082. 10.1177/1471301218798082. 10.1177/1471301218798082 30205692

[72] Beach SR, Kinnee E, Schulz R. Caregiving and Place: Combining Geographic Information System (GIS) and Survey Methods to Examine Neighborhood Context and Caregiver Outcomes. Innov Aging. 2019;3(3):igz025. 10.1093/geroni/igz025. 10.1093/geroni/igz025 PMC6735773 31528713

[73] Liu Y, Fu R, Roberto KA, Savla J (2019). Depressive symptoms among adult children caregivers in China: moderating effects of working status and gender. Aging Ment Health.

[74] Mosca I, van der Wees PJ, Mot ES, Wammes JJG, Jeurissen PPT (2017). Sustainability of long-term care: puzzling tasks ahead for policy-makers. Int J Health Policy Manag.

[75] OECD (2011). Policies to support family Carers. In: help wanted?: providing and paying for long-term care.

[76] Yghemonos S (2016). The importance of informal carers for primary health care. Prim Health Care Res Dev.

[77] Safwan Hamdy M, Md Yusuf M (2018). Review on public long-term care services for older people in Malaysia. MJoSHT..

